# Exploitation of Marine Molecules to Manage Alzheimer’s Disease

**DOI:** 10.3390/md19070373

**Published:** 2021-06-28

**Authors:** Marisa Silva, Paula Seijas, Paz Otero

**Affiliations:** 1 MARE—Marine and Environmental Sciences Centre, Faculty of Sciences, University of Lisbon, Campo Grande, 1749-016 Lisbon, Portugal; mpdsilva@fc.ul.pt; 2 Department of Plant Biology, Faculty of Science, University of Lisbon, Campo Grande, 1749-016 Lisbon, Portugal; 3 Department of Pharmacology, Faculty of Pharmacy, University of Santiago de Compostela, 15782 Santiago de Compostela, Spain; paula.seijas.villaverde@rai.usc.es; 4 Department of Production and Characterization of Novel Foods, Institute of Food Science Research (CIAL), Campus of International Excellence UAM+CSIC, 28049 Madrid, Spain; 5 Nutrition and Bromatology Group, CITACA, Faculty of Food Science and Technology, University of Vigo, Ourense Campus, 32004 Ourense, Spain

**Keywords:** Alzheimer’s disease, marine drugs, sponges, algae, yields, natural products

## Abstract

Neurodegenerative diseases are sociosanitary challenges of today, as a result of increased average life expectancy, with Alzheimer’s disease being one of the most prevalent. This pathology is characterized by brain impairment linked to a neurodegenerative process culminating in cognitive decline and behavioral disorders. Though the etiology of this pathology is still unknown, it is usually associated with the appearance of senile plaques and neurofibrillary tangles. The most used prophylaxis relies on anticholinesterase drugs and NMDA receptor antagonists, whose main action is to relieve symptoms and not to treat or prevent the disease. Currently, the scientific community is gathering efforts to disclose new natural compounds effective against Alzheimer’s disease and other neurodegenerative pathologies. Marine natural products have been shown to be promising candidates, and some have been proven to exert a high neuroprotection effect, constituting a large reservoir of potential drugs and nutraceutical agents. The present article attempts to describe the processes of extraction and isolation of bioactive compounds derived from sponges, algae, marine bacteria, invertebrates, crustaceans, and tunicates as drug candidates against AD, with a focus on the success of pharmacological activity in the process of finding new and effective drug compounds.

## 1. Introduction

Neurodegenerative diseases (NDs) are the XXI century paradigm, since they result from progressive loss of structure and/or function of neurons, sometimes leading to cell death [1]. One of the dominant factors for the escalation of these pathologies is the increase in the average life expectancy. Aging stands as a common factor to a diverse group of syndromes, and still few therapeutic advances towards finding a cure have been achieved. NDs represent the seventh leading cause of mortality worldwide, and their prevalence increases exponentially with age [2]. One of the most common NDs is Alzheimer’s disease (AD), which was first diagnosed and described by the German neurologist Dr. Alois Alzheimer in 1906 and is the most common cause of dementia in the elderly [3]. The term dementia is defined as a chronic progressive mental disorder that affects memory, thinking, understanding, and other essential brain functions [4]. This is characterized by a progressive loss of neurons, which results in brain atrophy mainly in the memory and learning areas of the brain. Consequently, motor, sensory, emotional, and cognitive alterations emerge [5]. Currently, there are approximately 50 million people who suffer from dementia worldwide, and this number is expected to increase to 66 million by 2030 and to 115 million by 2050 [6].

Alzheimer’s is characterized by the presence of amyloid-beta plaques and neurofibrillary tangles that are formed in the patient’s brain, and its pathogenesis is multifactorial. The origin of the disease is associated with the mutations in three major genes encoding amyloid precursor protein (APP) on chromosome 21, presenilin-1 (PS1) on chromosome 14, and presenilin-2 (PS2) [7]. The mutations in these genes lead to the formation of senile plaques in the extracellular region by amyloid-β protein (Aβ) and the hyperphosphorylation of tau protein that forms the neurofibrillary tangles intracellularly [8]. This causes widespread damage to nerve cells throughout the brain cortex, accompanied by early loss of cholinergic neurons from the basal region of the forebrain. The duration of AD is approximately from 8 to 10 years. In most cases, the patients who have been diagnosed with this disease are over 65 years old. The cause of AD occurrence is unknown; however, several hypotheses try to explain the development of this pathology [7]. The most important theories are described below.

The first hypothesis is the amyloid cascade. According to this hypothesis, the accumulation of amyloid-beta (amyloid plaques) triggers a cascade of events that lead to synaptic dysfunction, memory loss, and structural damage in the brain in the advanced stages of the disease [9]. Another theory is the cholinergic hypothesis, which is based on the significant loss of cholinergic signaling. This occurs due to a severe loss of white substance from the brain with a reduction in basal forebrain cholinergic neurons that can be observed in the postmortem cerebral cortex of Alzheimer’s patients [10]. The glutamatergic hypothesis is based on a gradual deterioration of adequate synaptic function through N-methyl-D-aspartate receptors that contain GluN2A and the development of excitotoxicity through NMDA receptors that contain GluN2B. If the activity of NMDA receptors is altered, amyloid-beta peptide can induce synaptic impairment, spinal loss, and neurodegeneration [11]. The mitochondrial hypothesis predicts that mitochondrial dysfunctions will trigger a deterioration of energy metabolism, producing an excess of reactive oxygen species, and consequently, will cause damage to the structure of DNA. The metabolic hypothesis assumes that mitochondrial dysregulation will increase the activity of oxidative phosphorylation [12]. The tau hypothesis is based on a tau dysfunction (abnormal levels, ubiquitination, or hyperphosphorylation) being sufficient to cause neuronal and synaptic loss. In AD, the tau protein is aggregated, forming paired helical filaments, and adopts a beta β-sheet conformation. These tangles are in areas where there is neuronal dysfunction, unlike senile plaques that appear in the brain of patients in a nonspecific and more general manner [13]. Another hypothesis is that involving protein kinase C, based on acquiring and modifying dendritic spines, in neuronal retraction and synaptic plasticity [14]. The elimination systems hypothesis is based on a failure in amyloid-beta elimination. The excessive accumulation of amyloid-beta peptides results in an imbalance between their production and their elimination in AD [15]. The neuroinflammation hypothesis is associated with an innate immune response characterized by the release of inflammatory mediators [16]. Finally, the cognitive reserve hypothesis explains the gap between brain aggression and pathological manifestations. It includes two elements: the brain (brain size, synaptic count, and dendritic branching) and the cognitive reserve (neuronal and compensatory reserve) [17].

**Figure 1:**
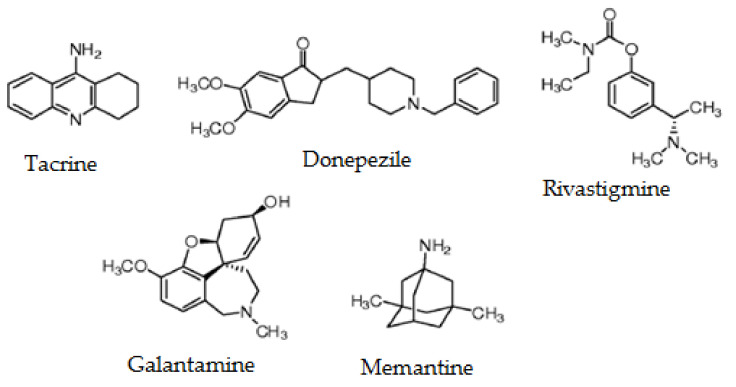
Chemical structures of FDA-approved drugs for Alzheimer’s disease treatment.

Currently, there are no effective pharmacotherapeutic agents to prevent or treat AD. The established therapy is only reliable to treat the symptoms of the pathology [18] by normalizing the levels of neurotransmitters that are affected in AD, i.e., with treatments producing an increase in acetylcholine levels (cholinesterase inhibitor drugs) [19] and a decrease in glutamate levels (glutamate inhibitor drugs) [20]. Figure 1 presents the main molecules currently used for AD treatment. The effectiveness of all these molecules varies between patients and stages of the disease, and in many cases, these drugs have adverse effects (nausea, vomiting, and diarrhea). For all these reasons, numerous efforts have been made in developing new therapeutic agents to find new drugs of value against AD [21,22,23,24,25,26,27]. It is widely known that the development of AD is linked to amyloid-β peptides and tau aggregation. Despite this, several other processes could contribute to the advance of AD, and some of them could be related to protein–protein interactions [28]. In this field, new research in AD therapy highlights the need for modulating protein–protein interactions and the important role of peptides as potential drugs in the context of the cross-interactions, due to the selectivity and affinity of these molecules [28]. In addition, studies suggest that the intracellular accumulation of tau is a key factor in the memory deficits and the neurodegeneration in AD; therefore, the development of drugs aimed at tau clearance has gained attention in recent years [29]. Targeted protein degradation using proteolysis-targeting chimeras (PROTACs) to degrade specific proteins from within cells represents a promising novel therapeutic strategy for AD and the related tauopathies [30]. As PROTACs could potentially be generated for any protein, they may be used to target tau, a natively unfolded undruggable protein. In addition, recent AD therapeutic approaches have focused on marine drug discovery. Marine organisms are capable of synthesizing different metabolites that are used to immobilize and capture prey, in addition to allowing them to defend themselves from predators [31]. These compounds range from small peptides and enzymes to highly complex secondary metabolites that show bioactivities in physiological systems such as those related to movement, circulation, and respiration. The importance of marine drugs against neurodegeneration is widely recognized [32,33]. In fact, some natural compounds are already in preclinical studies for AD treatment [34]. The main aim of this review is to describe the marine molecules that have the potential to become leaders in innovative drug discovery in AD as well as their isolation and the main sources for their obtention. Oceans serve as the habitat of a great diversity of organisms that live in different habitats characterized by specific physical and chemical properties such as salt and oxygen concentrations, pressure, radiation, temperature, and ocean currents.

## 2. Marine Organisms Producing Compounds for Neuroprotection

The oceans are the cradle of life and harbor a vast variety of marine organisms, covering 70% of the earth’s extension, being a source of primary materials and bioactive compounds with diverse applications [35,36]. More than 10,000 natural products of potential biotechnological interest have been isolated from marine organisms, mainly from organisms belonging to the base of the food chain (bacteria, fungi, micro- and macroalgae, gorgonians, sponges, nudibranchs, bryozoans, sea cucumbers, tunicates, and sea hares, among other marine organisms) [37]. These bioactive compounds result from the necessary adaptations that these organisms need to survive in this “primordial soup”, as secondary metabolites used in defensive/offensive mechanisms [38]. Currently, 38% of marine compounds reported as having pharmacological activity come from microorganisms such as bacteria, protozoa, fungi, and microalgae [31]. Approximately 20% are obtained from marine sponges and corals, with special emphasis on the Porifera phylum, being macroalgae, fish, mollusks, echinoderms, and coelenterates, with fewer reports on this theme [39]. In this section, the main marine organisms reported as producers of novel neuroprotection molecules will be described in crescent order of complexity.

### 2.1. Bacterioplankton

Bacteria were the primordial life forms on earth and are ubiquitous in every type of environment, even extreme ones [40]. Marine bacteria are unicellular prokaryotes, ranging from 0.5–1 µm in size with diverse shapes and arrangements, and are considered the smallest autonomous sea organisms known [41]. They can be found as free-living or in symbiosis/parasitism relations with more complex organisms (e.g., corals, sponges, or macroalgae), and it is estimated that the oceans harbor about 10,000,000 different bacterial taxa [42].

Regarding bioactive molecules, their terrestrial counterparts have been extensively explored, as 50% of antibiotics known are derived from them, representing an area considered in decline today [43]. For this reason, bacterioplankton represent a new field of exploration and are often described as “chemical gold”, since they are considered to be an excellent source of new unexplored therapeutics with distinct applications [43]. Marine bacterial flora also has advantages in biotechnological terms, since its mass production proves to be more sustainable, more environmentally friendly, and cheaper through large-scale production and fermentation processes compared to more complex organisms [44,45,46]. Due to its rapid growth and the use of cheap carbon sources, its production and industrialization are relatively advanced, involving subsequent steps described as follows. Initially, microorganisms are isolated from the natural environment using shotgun or objective approaches [44,45,47,48,49,50,51]. After sample collection, an appropriate culture medium for the growth of the sampled community is chosen or developed, and then subsequent isolation of pure cultures and screening of desired properties is performed [46]. Selected strains can also be improved through genetic and metabolomic engineering methodologies by identifying and introducing the gene expression sequence of the desired bioactive compound into an engineered host-microbe (e.g., *S. cerevisiae* or *E. coli*), bringing together genetic elements from two different genomes into one unit, achieving in this way the fermentation rates necessary for industrial pharmaceutical production [52]. Moreover, the heterologous expression systems combined with modern metagenomics make the possibility of tracing and discovering new bioactive compounds in bacterioplankton considered uncultivable real by giving prior access to the target microbiome genome using next-generation sequencing approaches, elaborating metagenomic libraries without the need to cultivate the microorganism in question [53,54].

### 2.2. Marine Microalgae

Marine microalgae are responsible for the primary production of ecosystems as eukaryotic photosynthetic beings [55]. They live freely in the water column or sediments or as photosymbionts with more complex organisms (e.g., sponges, corals, mollusks) [56,57]. Phytoplankton is responsible for the production of half of the atmospheric oxygen and has the capacity to use light and inorganic compounds and convert them into complex macromolecules (lipids, pigments, proteins, polysaccharides, etc.) [28]. It is estimated that there are 50,000 different species of phytoplankton that can be divided into three groups: Chlorophyta (green algae), Chrysophyta (golden-brown algae, yellow algae, and diatoms), and Pyrrhophyta (dinoflagellates), with different shapes and sizes ranging from 2 to 200 µm [58,59,60,61]. As many microalgal species have short reproductive cycles, considerable amounts can be produced in closed systems, i.e., photobioreactors under controlled conditions [62,63,64]. However, the biosynthesis of valuable metabolites in considerable amounts is achieved in response to changes in environmental conditions such as nutrient availability, salinity, light, temperature, and pH. A physiological reaction to a stressor translates into a modulation in their metabolism to adapt and survive. Therefore, changes in the culturing conditions of microalgae consequently lead to an alteration in their biochemical composition that can enhance the production of target molecules of interest [65,66,67,68,69]. In this sense, photobioreactors present themselves as excellent cultivation systems to improve and refine control conditions and cultivation methods, enhancing and stimulating the production of new bioactive compounds of interest [64,69]. Though this is promising, microalgae present some challenges, namely in terms of taxonomic identification and the fact that only a few microalgae genomes are fully sequenced and known today [60,61,70,71]. Despite their brief reproductive cycles, many phytoplankton species are fastidious with slow growth under controlled laboratory conditions [46]. Although the number of novel bioactive compounds of phytoplankton origin has increased in recent years, there is still much more to unravel, due to the challenging task regarding description and identification of phytoplankton and the optimization and enhancement of culture conditions to make them a sustainable and appealing source of supply [60,61,70,71,72].

#### Marine Macroalgae

Marine macroalgae, more commonly known as seaweeds, are eukaryotic multicellular photosynthetic organisms that inhabit intertidal and tropical environments [73]. Based on pigment abundance, they are classified into three distinct classes: green algae (Chlorophyceae), brown algae (Phaeophyceae), and red algae (Rhodophyceae) [73]. Marine macroalgae are well known for their use in the food industry, especially in Asian countries, though their application regarding healthcare has been connected to traditional medicine [74,75]. So far, more than 32,000 novel compounds have been reported from macroalgae’s vast array of bioactivities and applications [76,77,78] Regarding the extraction of these compounds, new green approaches have gained strength in recent years. They are characterized by the reduction in the number and amount of solvents, faster extraction times, good performance at low temperatures, good selectivity towards the targeted compound, and prevention of undesired reactions and byproducts during extraction, having for these reasons gained more followers compared to traditional extraction techniques [79]. Among these green innovative extraction techniques applied in the isolation of bioactive compounds from marine macroalgae are microwave-assisted extraction (MAE), ultrasound-assisted extraction (UAE), pressurized liquid extraction (PLE), and supercritical fluid extraction (SCFE) [80,81,82]. UAE is based on the application of ultrasound waves above 20 kHz to create bubbles and zones of high and low pressure [83]. MAE uses microwaves to heat the samples, evaporating the intracellular fluids [84,85]. PLE is a technique based on the use of conventional solvents at temperatures and pressures high enough to maintain the solvent in the liquid state during the whole extraction procedure [86,87]. Finally, SCFE uses a supercritical fluid as a solvent. It is based on the principle of extraction with fluids in their supercritical conditions, where temperature and pressure are raised above their critical point, with characteristics of both liquids and gases. However, the exploration of necessary macroalgae to conduct clinical trials can have a huge impact on marine ecosystems’ sustainability. Moreover, the composition of bioactive compounds has species-specific variation and is dependent on several parameters such as organism age and size, predation pressure, tissue type, and abiotic factors [88]. The development of efficient culture approaches is mandatory to prevent pressure and overexploitation of natural populations [88].

### 2.3. Marine Sponges

Marine sponges are multicellular invertebrate sessile organisms that belong to the Porifera phylum; they have a wide distribution, being able to colonize different kinds of habitats in terms of temperature, salinity, and pressure [89]. As their name implies, these organisms have a soft body composed of cell layers that house the spicule skeleton, composed of a protein known as spongin, calcium carbonate, and silica, and the identification of these animals is determined by characterizing the size and shape of these spicules [90,91]. So far, approximately 15,000 different species have been cataloged [91]. Sponges are sessile filter feeders; their simple structure of channels allows them to feed on the microorganisms and matter in water that circulates through their bodies. However, this particular condition also exposes them to potentially hazardous particles, infectious agents, and predators, forcing them to cope with these external threats by developing strong chemical defense mechanisms [91].

Here the story of new marine bioactive compounds begins in 1950 when spongothymidine and spongouridine, nucleosides that led to the synthesis of the first antiviral drug, were primarily isolated from *T. crypta*, a tropical specimen [92]. Since then, hundreds of new chemically diverse bioactive compounds have been reported from several sponge genera [93]. Sponges are reported to synthesize a wide range of compounds, including nucleosides, alkaloids, cyclic peptides, sterols, and terpenes [94]. Nevertheless, the credit for the new compounds cannot be given to sponges alone, since they are symbiont beings, sheltering a microbial flora that provides a chemical defense to its host by the production of secondary metabolites. Thus, most of the discoveries in sponges on this theme have origin in their symbionts [95]. This is not discouraging, since the conditions for cultivation and maintenance of these animals are challenging and need improvement, and it is not environmentally sustainable to obtain specimens from nature to feed this type of research. Therefore, the discovery of new bioactive compounds through sponges can rely on metagenomic methods described above to obtain crude extracts that can be fragmented and purified with aid from the new green approaches previously mentioned [96].

### 2.4. Marine Tunicates

Tunicates, also known as ascidians belong to the Urochordate subphylum, are characterized by their vertebrate-like larval free stage contrasting with their sessile invertebrate adult stage [97]. Tunicates have sexual and asexual reproduction and have varied phenotypes; they are also the only animals known for the production of cellulose, and up to now about 3000 species have been described [97]. The drug discovery in ascidians began in the 1960s with the discovery of the antiviral and antitumor geranyl hydroquinone, isolated from the tropical genus *Trididenium* [98]. Since then, 1200 natural compounds have been identified from this subphylum [99]. As mentioned, tunicates are sessile in their mature stage and have been reported to be the home of a diverse microbial community [100,101]. In this sense, some of the new compounds of biotechnological interest first identified in tunicates were later reported as originating from their associated symbiotic flora [102,103]. Techniques such as highly advanced analytical tools, new methods for genetic and chemical dereplication, molecular biology tools, LC-MS, and NMR metabolomics approaches, combined with efforts of computational biology, have directed and increased the high-throughput screening efficiency of exploring novel therapeutics in tunicates [104,105,106,107].

### 2.5. Marine Arthropods

Crustaceans are a large arthropod subphylum, consisting of more than 52,000 species [108]. They are the oldest arthropods, from which insects evolved, and are characterized by their chitinous exoskeleton [108]. Crustaceans are considered a valuable nutritional source and play a huge economical role in fishery and aquaculture industries today [109,110]. The exploration of bioactive compounds from this subphylum is quite recent and motivated by the use of food waste. One of the first approaches occurred in 2013 with the purification of a peptide (CMCC-1) with antimicrobial activity [111]. Subsequently, several studies followed this trend mainly in the study of the main constituent of the exoskeleton of these arthropods, chitin, due to its abundance in the remains of the food industry, bioavailability, structural stability, and safe handling [112]. Chitin is relatively simple to extract with a four-step approach that comprises demineralization, deproteinization, decolorization, and oxidation [113]. From it is derived one of the most popular molecules with crustacean origin, chitosan, which will be discussed in more detail in the following sections. The use of marine-based foods remains a sustainable alternative, still little explored, for the search for new bioactive compounds and should be stimulated.

## 3. Marine Compounds against Alzheimer’s Disease: From Sea to Cells

The continuously growing interest in marine-derived molecules is justified by the structural properties that are not usually found in terrestrial organisms, exhibiting considerable bioactivity, up to ten times higher than that of terrestrial-sourced compounds [22]. In this section, we describe the mechanisms of action of the main compounds isolated from marine organisms with neuroprotection activity against AD, as summarized in Table 1.

### 3.1. Gracilins

**Figure 2:**
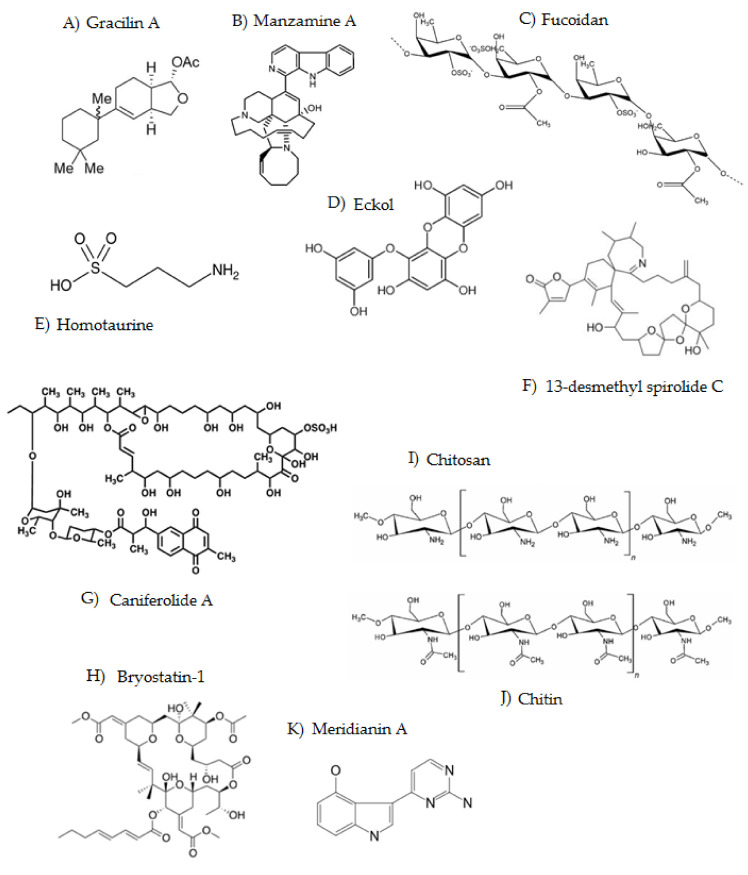
Chemical structure of marine molecules with neuroprotective effects against Alzheimer’s disease: gracillin A (**A**), manzamine A (**B**), fucoidan (**C**), eckol (**D**), homotaurine (**E**), 13-desmethyl spirolide C (**F**), caniferolide A (**G**), bryostatin-1 (**H**), chitosan (**I**), chitin (**J**) and meridianin A (**K**).

Gracilins are sponge-derived diterpenoids isolated from marine sponges. The first analog structurally elucidated was gracilin A (Figure 2A), discovered in 1985 and isolated from the Mediterranean sponge *Spongionella gracilis* [124]. This was followed by the isolation of gracilin B and gracilins G–I from *Spongionella pulchella* [125] and gracilins J–L [39]. Gracilin A was described to have the most potent immunosuppressive and neuroprotective activity of all analogs. All of them have in their structure the group diacetoxyhexahydrodifuro [2,3-b;3,2-d]furan-2-one, a moiety that is uncommon in nature [27]. These compounds can inhibit the beta-secretase 1 enzyme, the kinase enzyme regulated by extracellular signals, and reduce the hyperphosphorylation of the tau protein [27]. Gracilins generate a neuroprotective effect on primary neurons with restored mitochondrial membrane potential and inhibition of alterations produced by the classical uncoupler of mitochondrial oxidative phosphorylation carbonylcyanide-p-trifluoromethoxyphenylhydrazone [126]. Moreover, they can enhance the expression of nuclear factor erythroid 2-related factor 2 (NRF2), which is involved in the activation of the antioxidant pathway. The antioxidant properties are responsible for the specific interactions of gracilins with the jaws proteins which are involved in apoptosis or the Nrf2-Keap1-ARE signaling pathway, ensuring their mechanism of action on Nrf2 levels in the nucleus [123]. In addition, gracilins have an important anti-inflammatory action, due to their ability to inhibit the enzyme phospholipase A2 (PLA2), which can reduce the production of reactive oxygen species (ROS) induced by amyloid-β [127].

### 3.2. Manzamines

Manzamines are alkaloids characterized by the presence of a β-carboline structure attached to a pentacyclic diamine ring, having 8- and 13-membered rings on a pyrrolo[2,3-i]isoquinoline framework [128]. Manzamine A (Figure 2B) was isolated for the first time from the marine sponge *Haliclona* sp. in 1986 [129]. Its activity to inhibit the GSK3beta enzyme noncompetitively was later discovered by in vitro studies when it was observed that an increase in the concentration of ATP did not cause a change in its inhibitory activity. Today, more than 100 analogs have been characterized, and it is likely that many more remain to be tested in medicinal chemistry studies [130]. Manzamine A can bind to a pocket close to the catalytic region of GSK3beta, inhibiting the enzyme allosterically [114]. The inhibitory capacity of manzamines on GSK3beta and other kinases was evaluated in a study carried out in human neuroblastic cells, and it was found that manzamine A is the only analog capable of inhibiting GSK3beta and CDK5. Likewise, the treatment of SH-SY5S cell cultures with different concentrations of manzamine A resulted in a decrease in the phosphorylation of the epitope Ser 396 of tau, which is specifically phosphorylated by GSK3beta [114].

### 3.3. Fucoidan

Fucoidan (Figure 2C) is a sulfated polysaccharide-type compound extracted from brown seaweeds [131]. Structurally it is composed of a backbone of alpha(1→3)-L-fucopyranose or L-fucopyranosyl residues linked to alternate alpha(1→3) and alpha(1→4). Fucoidan is reported to act on different stages of the inflammatory process [132]. It blocks the lymphocyte adhesion and invasion, inhibits the multiple enzymes, and induces apoptosis [132]. Fucoidan can block the activation of the enzymes caspase-9 and caspase-3, suggesting that the inhibition of neuronal death by this compound mainly occurs through the inhibition of apoptosis since these enzymes play an important role in apoptosis processes [115]. A fucoidan treatment has been found capable of reducing the inhibitory effect of amyloid-beta on protein kinase C (PKC) phosphorylation [133]. Fucoidan can prevent amyloid-beta neurotoxicity and stimulate neuronal survival. Protein kinase C produces an inactivation of GSK-3b and, in turn, an accumulation of cytoplasmic beta-catenin in the nucleus which causes the TCF/LEF-1-dependent transcriptional activation of genes that are related to growth and the differentiation that is needed to stimulate the survival of neurons [133]. Recently, it was also demonstrated that fucoidan is a neuroprotective agent in a mouse model of AD [21]. The compound showed neuroprotective effects against Aβ- and D-Gal-induced apoptosis in PC12 cells and D-Gal-induced learning and memory impairment in AD model mice. The related mechanisms comprise the regulation of the cholinergic system, oxidative stress reduction, and the inhibition of the caspase and mitochondria apoptosis pathways [21].

### 3.4. Phlorotannins

Phlorotannins are polyphenols that can be found in the brown algae species *Ecklonia cava, Ecklonia stolonifera,* and *Eisenia bicyclis* [130]. Phlorotannins such as phlorogucinol, eckol, dieckol, phloroeckol, and phlorofurofucoeckol are related to an increase in the most important central neurotransmitter in the brain, the neurotransmitter acetylcholine, by inhibiting the activity of the enzymes acetylcholinesterase and butyrylcholinesterase. The structure of eckol is represented in Figure 2D.

Eckol, dieckol, and 8,8′-bieckol from Ecklonia cava can inhibit the BACE-1 enzyme [116]. The drugs currently used for the AD treatment mainly act by inhibiting the enzyme acetylcholinesterase, so the discovery of the inhibition of the BACE-1 enzyme by phlorotannin may improve the drugs and treatment for patients affected by AD. Recently, it was shown for the first time that the phlorotannin dieckol regulates APP proteolytic processing and Aβ production via the regulation of the PI3K/Akt/GSK-3 β signaling pathway [134]. Moreover, the addition of LY294002 counteracts all the effects of dieckol, demonstrating that Akt/GSK-3β is the primary pathway mediating Aβ production in SweAPP N2a cells.

### 3.5. Homotaurine

Homotaurine (3-aminopropanesulfonate) (Figure 2E) is a small natural amino sulfonate compound originally extracted from different species of marine red algae and then chemically synthesized and used in clinical use under the name of tramiprosate [135]. The molecule was called homotaurine due to its high homology with taurine (2-aminoethanesulfonate), which is an important amino acid in various metabolic processes of the body. The two molecules share a very similar structure, although homotaurine contains one additional carbon. The therapeutic efficacy of homotaurine in AD has been investigated in three phase II and three phase III clinical studies, providing a relevant neuroprotective effect by its specific antiamyloid activity and by its γ-aminobutyric acid type A receptor affinity [117]. This compound acts as a modulator of excitatory neurotransmission since it exhibits affinity for GABA receptors [136]. A therapeutic mechanism showed that tramiprosate works by blocking the production of neurotoxic amyloid-β oligomers by coating the amyloid peptide to prevent its misfolding. The amyloid-β oligomers are believed to be a key pathogenic driver in the disease process. The homotaurine mechanism prevents the self-assembly of misfolded proteins into amyloid-β oligomers that leads to amyloid aggregation and subsequently causes neurotoxicity and clinical progression of AD [137]. This genetic knowledge emerged after phase III clinical studies of tramiprosate in AD patients and prompted a precision medicine approach and analysis of the genetically defined subset of AD patients in homotaurine studies. The knowledge of this new molecular mechanism and the evaluation of genetically defined clinical data sets have opened new opportunities to optimize tramiprosate and its derivatives for the treatment of Alzheimer’s.

### 3.6. Spirolides

Spirolides belong to the family of cyclic imines, an emerging group of lipophilic marine toxins produced by the dinoflagellates *Alexandrium ostenfeldii* and *Alexandrium peruviaunum* [138]. Structurally, they are macrocyclic compounds with imine and spiro-linked ether moieties [139]. Their mode of action is based on the interaction with muscle-type and neuronal nicotinic acetylcholine (ACh) receptors (nAChR); however, no human intoxication has been reported, since they present low oral toxicity [139,140,141]. The main representative from this group is 13-desmethyl spirolide C (13-desMeC) (Figure 2F). It induced positive effects on AD markers, with an increase in N-acetyl aspartate (NAA) levels. An in vivo study showed an increase in the synaptophysin levels and also a decrease in the intracellular amyloid-beta levels in the hippocampus of treated 3xTg-AD [26]. 13-Desmethyl spirolide C can cross the blood–brain barrier and provide beneficial effects against AD [26].

### 3.7. Caniferolide A

Caniferolide A is a macrolide-type compound isolated from extracts of a marine culture of *Streptomyces caniferus*, a Gram-positive bacterium included in the phylum Actinobacteria [142]. Studies show that this compound alleviates the distinctive characteristics of AD [25]. Caniferolide A (Figure 2G) leads to the reduction in neuroinflammatory markers in BV2 glial cells activated with lipopolysaccharide (LPS), being able to block the translocation of FFKB-p65 to the nucleus and activate the Nrf2 pathway [25]. It also was found to cause decreases in proinflammatory cytokines (IL-1b, IL-6, and TNF-alpha), reactive oxygen species (ROS), and the release of nitric oxide (NO) and inhibit the activities of iNOS, JNK, and p38. In addition, the compound blocks the activity of the BACE-1 enzyme and attenuates amyloid-beta activation of the microglia by drastically decreasing the levels of reactive oxygen species. The phosphorylated state of the tau protein was evaluated in SH-SY5Y tau 441 cells. Caniferolide A reduced the phosphorylation of Thr212 and Ser 214 by targeting the p38 and JNK MAPK kinases. On the other hand, the antioxidant properties of the macrolide were determined in an oxidative stress model with SH-SY5Y cells treated with hydrogen peroxide [25]. The compound decreased ROS levels and increased cell viability and GSH content by activating the nuclear factor Nrf2. Finally, the neuroprotective capacity of the compound was confirmed in two transwell coculture systems with activated BV2 cells (both lipopolysaccharide and amyloid-beta) and transfected and wild-type SH-SY5Y cells. The addition of caniferolide A to microglial cells produced a significant increase in neuroblastoma survival in both cases. These results indicate that the compound can target many pathological markers of AD, suggesting that is a very interesting drug for a polypharmacological approach to the disease.

### 3.8. Bryostatins

Bryostatins are a group of macrolides characterized by a highly oxygenated structure with 11 chiral centers [119]. At least 21 bryostatins have been isolated and elucidated to date, although bryostatin-1 is the most investigated analog [143]. Bryostatin-1 (Figure 2H) was initially isolated from the marine invertebrate organism *Bugula neritina* (a brown bryozoan). Bryostatins act as potent modulators of protein kinase C (PKC). Although bryostatins are hydrophilic molecules, they bind strongly to PKC, with a potency similar to that of phorbol ester [119]. Bryostatin-1 induces a fast short activation and self-phosphorylation of PKCs that consecutively induces PKC membrane translocation with successive PKC downregulation. Bryostatins were studied in clinical trials as agents against cancer, against AIDS/HIV, and in patients with AD [120]. For this reason, in recent years, these compounds have become considered pharmaceutically promising by various research groups.

### 3.9. Chitosan

Chitosan (Figure 2I) is a polysaccharide from marine organisms formed by the partial deacetylation of chitin (Figure 2J), which is present in the exoskeletons of crustaceans and insects, as well as in the cell walls of some fungi [112]. It comprises copolymers of aminosaccharides of beta-(1–4)-D-glucosamine and N-acetyl-D-glucosamine obtained by the hydrolysis of acetyl groups (H_3_C-CO) [112]. This process liberates the primary amino groups (R-NH_2_) and gives chitosan a cationic nature. Chitosan has poor solubility. To increase the applications in biomedical applications, chitosan is transformed into chitosan oligosaccharides, molecules easily soluble in water [144]. Activities linked to chitosan and chitosan oligosaccharides include antioxidant, antibacterial, anti-HIV, anti-inflammatory, antidiabetic, and immunomodulatory [145]. In AD, chitosan has a protective effect; it controls the level of amyloid-beta through the expression and activity of the BACE-1 enzyme. Chito-oligosaccharides are also used as inhibitors of the enzyme acetylcholinesterase and have shown beneficial effects in preventing AD [121]. Chitosan and its derivatives have shown neuroprotective properties such as suppression of amyloid-β formation, AChEI activity, anti-neuroinflammatory activity, and apoptosis inhibition. Up to now, most neuroprotective activities of chitosan and its derivatives have been observed in vitro [146].

### 3.10. Meridianins

Meridianins are indole alkaloids initially obtained from *Aplidium meridianum* tunicates collected in the South Atlantic [147], although some meridianins have also been obtained from tunicates of genus *Synoicum* in the Arctic [148]. Studies in silico and in vivo showed these compounds are capable of binding to tau-specific protein kinases such as GSK3beta and CK1sigma and with dual specificity to CLK1 and DYRK1A [122]. Among the meridianin family, meridianin E was found to be the most potent inhibitor of protein kinases CDK1/B, CDK5/p25, GSK3a, GSK3b, and CK1 [123]. Figure 2K shows the structure of meridianin A.

## 4. Isolation of High-Value Molecules with Neuroprotective Effects against Alzheimer’s Disease

The marine habitat is not only a productive source for the discovery of novel entities but also a tool to identify new cellular targets for therapeutic intervention [149]. The marine ecosystem is an important source for drug development against AD. However, the amounts of bioactive natural products in these organisms are always low. Today, it is very crucial to develop effective and selective methods for the extraction and isolation of those bioactive natural products [150] since the pharmacological activity in vitro and in vivo has to be checked after the purification of these molecules [151]. This section describes the variety of methods and protocols used in the extraction and isolation of marine natural products against AD and the main sources for drug development. The information is summarized in Table 2.

### 4.1. Gracilins

Gracilins can be obtained from marine natural sources or by synthesis [125,152]. The sponges *Sponginella gracilis* and *Spongionella pulchella* are the only gracillin producer species described so far [124]. There are not many studies regarding the isolation of gracilins from natural sources. One study described the purification of 152 mg of gracilins H and gracilins J–L from Philippines cultures of *Spongionella* spp. using a modified Kupchan scheme [39]. For the isolation, 5 g of the crude extract of *Sponginella* sp. was fractionated in hexane and dichloromethane. Afterward, the combined extracts were cleaned using a Sephadex LH-20 column equilibrated with methanol/dichloromethane (1:1) and purified by reversed-phase HPLC using a gradient of acetonitrile in H_2_O as eluent (50–100% over 50 min, 100% for 10 min) at a flow rate of 1.25 mL/min. Finally, 9 mg of gracillin J, 11 mg of gracillin K, 17 mg of gracillin L, and 115 mg of a mixture of gracillin H and a gracilin derivative were obtained [39].

### 4.2. Manzamines

About 100 manzamine alkaloids have been isolated from more than 16 species of marine sponges belonging to 10 genera (*Amphimedon, Pachypellina, Haliclona, Reniera, Cribochalina, Petrosia, Ircinia, Hyrtios, Acanthostrongylophora,* and *Xestospongia*) and belonging to five families (Chalinidae, Niphatidae, Petrosiidae, Thorectidae, Irciniidae). These sponges are found in Okinawa, the Philippines, Indonesia, the Red Sea, Italy, South Africa, and Papua New Guinea [128,129]. AlTarabeen and colleages purified seven manzamines (ircinal E, manzamine A, 8-hydroxymanzamine A, manzamine F, manzamine A N-oxide, 3,4-dihydromanzamine A N-oxide, and nakadomarin A) from *Acanthostrongylophora ingens* collected at Ambon, Indonesia [153]. The amount of 1.3 kg of the dried sponge was extracted with methanol and subjected to solvent–solvent partitioning to give EtOAc and n-BuOH fractions [154]. Afterward, a clean-up was performed with Diaion HP-20 and Sephadex LH-20, followed by purification with semipreparative reversed-phase HPLC using a gradient of methanol/water/TFA and a flow rate of 5 mL/min. The resulting pure amounts of manzamines were 21.2 mg (5 mg manzamine A, 5 mg 8-hydroxymanzamine A, 9 mg manzamine F, 1.1 mg manzamine A N-oxide, and 1.1 mg 3,4-dihydromanzamine a N-oxide) [153,154], i.e., a total of 16.92 mg/kg of dried biomass.

### 4.3. Fucoidan

Fucoidan is mainly obtained from brown macroalgae such as *Fucus vesiculosus*, *Undaria pinnatifida, Laminaria japonica, Spirarea japonica, Nizamuddinia zanardinii,* and several *Sargassum* spp. [83,155,156,157,158]. It is a polysaccharide composed of several monosaccharides, such as galactose, xylose, arabinose, mannose, glucose, or glucuronic acid, although fucose is the major monomer [159]. Generally, fucose content is around 40% of the total monosaccharides of fucoidan, but in some species, it may rise to 80% [155]. Fucoidans are found as part of algae cellular walls, so extraction techniques are essential to obtain rich extracts of these bioactive molecules. Traditionally, hot water has been the most used method; however, in recent years, innovative extraction techniques have arisen to recover fucoidans more efficiently and cleanly, usually followed by further purification and fractionation procedures [160,161]. The structure of fucoidan is usually highly heterogeneous and varies according to algae species and the applied extraction methods as the latter may also hydrolyze certain residues in diverse patterns [84]. Today, the methods for the extraction of fucoidans from seaweeds include solid–liquid (S-L) extraction, sample conditioning, pre-extraction, conventional extraction, and alternative extraction procedures [157]. Among innovative techniques for fucoidans extraction, some of the most employed currently are UAE, MAE, PLE, and SCFE [84,85,86,87,157,162]. Using UAE, the amount of 3.51 mg fucoidan/100 mg DW was successfully extracted from *Nizamuddinia* *zanardinii* [83]. In addition, it was possible to extract 8.23 mg fucoidan/100 mg *Spirarea japonica* (DW) using PLE with 0.1% NaOH at 140 °C and 50 bar of pressure [158]. Extracts obtained by these extraction techniques are complex mixtures composed mainly of polysaccharides, proteins, and polyphenols. To obtain purified fucoidan fractions, further purification processes are also necessary, including protein precipitation; liquid–liquid fractionation; uronic acid removal by calcium chloride treatment; membrane filtration; and anionic, size-exclusion, or affinity chromatography [157].

### 4.4. Phlorotannin

Phlorotannins are common tannins that can be found in a variety of brown macroalgae species of *Sargassum*, *Ecklonia*, and *Fucus* (*Saragassum fusiforme, Sargasum muticuma, Fucus vesiculosus, Eclonia maxima*, etc.) [163,164,165,166,167,168], and, like fucoidans, they are not present in terrestrial plants [169]. Phlorotannins are constituted of molecules of phloroglucinol that polymerize with ease between C1 and C3. They are grouped into three distinctive classes based on the coupling between subunits: fucols, phloroetols, and fucophloroteols. The complexity of their structure is correlated with the increase in phloroglucinol subunits (three to seven subunits) [170]. Phlorotannins are easily extracted from powder algae with a solid–liquid extraction with organic solvents such as ethanol 30%, ethyl acetate, and acetone 67% (*v*/*v*) at 25 °C. [165,166]. To maximize the extraction of phlorotannins from seaweeds, nonconventional extraction techniques such as MAE [171], UAE [172] and PLE [167] have been used. After extraction, their purification can be performed by a clean-up with Sephadex LH-20 size-exclusion chromatography and by high-speed counter-current chromatography (HSCCC) using a solvent system of n-hexane/ethyl acetate/methanol/water (2:8:3:7, *v*/*v*/*v*/*v*) [168]. Using all these conditions, phlorotannin yields in seaweeds ranged from 0.3 to 6.36% [165,166,167,168,171,172].

### 4.5. Homotaurine

Homotaurine is found in several marine organisms from which it could be obtained. This molecule has been documented in red algae (*Botryocladia leptopoda, Acanthophora spicifera, Gelidium micropterum, Hypnea boergeseniid, Gracilaria corticate, Gracilaria arcuate,* *Gracilaria pygmaea,* *Grateloupia livida, Chondrus ocellatus, Rhodymenia palmata, Acrosorium uncinatum,* *Actinotrichia fragilis, Helminthocladia australis*), green algae (*Caulerpa racemosa,* *Cladophora densa,* *Ulva fasciata*), brown algae (*Cystoseira indica,* *Cystoseira trinodis, Sargassum tenerrimum, Dictyota dichotoma, Cystoseira myrica, Iyengaria stellate, Padina boergesenii*), and unicellular green algae (prasinophytes such as *Ostreococcus* and *Micromonas*) [173,174,175]. Recently, it was found for the first time in bacteria [176]. Among these species, the higher amounts of homotaurine are found in the red algae species *H. boergesenii*, *G. corticate*, and *G. pygmaea* with amounts of 702, 475, and 333 µg/g, respectively. The research about the extraction and purification of homotaurine is scarce, although some studies pointed out that the extraction of homotaurine from algae can be easily performed with ethanol 70–80% at room temperature stirring for 12 h [173,174,175].

### 4.6. Spirolides

Spirolides consist of many molecules, although 13-desmethyl spirolide C is the most frequent and the most interesting from a pharmacological point of view [140]. 13-Desmethyl spirolide C, 13,19-didesmethyl spirolide C, and 20-methyl spirolide C are the only pure molecules commercially available [177]. The availability of spirolides throughout the world has been a long-term problem due to the difficulty of obtaining them by extraction from mollusks or the difficulty of their synthesis. Although these compounds can be also found in edible species (mussels, clams, cockles), in gastropods (*Gibbula umbilicalis, Nucella lapillus, Patella intermedia, Monodonta* sp.), and starfish (*Marthasterias glacialis*) [178,179], the best way to obtain them is from the dinoflagellates producing spirolides. Up to now, the only described producer is *Alexamdrium ostenfeldii/Alexandrium peruvianum*, and the type and amount of spirolides produced by the dinoflagellates can vary according to strains from different locations [138]. Otero and coworkers developed a method to purify two spirolides (13-desmethyl spirolide C and 13,19-didesmethyl spirolide C) from cultures of *Alexandrium ostenfeldii* [180]. The dinoflagellates grew in batches under controlled conditions of salinity, light, and temperature. The protocol designed presented several stages of extraction, separation, clean-up in a solid-phase extraction, and purification by preparative high-performance liquid chromatography system coupled to a mass spectrometer detector. Using this protocol, they were able to purify around 1.15 mg of spirolides with a purity higher than 97% [180].

### 4.7. Caniferolide

Caniferolides are new bioactive glycosylated 36-membered polyol macrolides from *Streptomyces caniferus*, a marine-derived Gram-positive bacterium included in the phylum Actinobacteria [142]. One study describes the extraction of caniferolides A–D from cells of *Streptomyces caniferus* CA-271066 (1 L of culture) with ethyl acetate and further isolation by Sephadex LH-20 chromatography with methanol and dichloromethane as eluent and a semipreparative reversed-phase HPLC (Agilent Zorbax RX-C8, 9.4 mm × 250 mm, 7 μm; 3 mL/min) with a linear gradient of CH_3_CN/H_2_O from 40 to 50% CH_3_CN in 40 min. The purified amounts were 10.0 mg caniferolide A, 3.6 mg caniferolide B, and 4 mg caniferolide C [25,181]. In parallel, caniferolide D was purified after an extra step of reversed-phase semipreparative HPLC (column XBridge C-18, 10 × 150 mm) with a linear gradient of CH_3_CN/H_2_O from 50 to 60% CH_3_CN over 30 min to yield 1 mg [181]. In addition, caniferolide C was isolated from the bacterium Streptomyces sp. ISID311 (1L of culture) by purifying the crude extract (2.3 g) after a partition of ethyl acetate and water. First, an SPE-C18 clean-up (55 μm, 20 g) was used, and then a semipreparative HPLC with a C18 semipreparative column and gradient of MeOH and H_2_O (0.1% of acetic acid) was used to yield 14.2 mg of caniferolide C [182].

### 4.8. Bryostatins

Bryostatin-1, discovered in the 1960s, was initially isolated from the extract of *Bugula neritina* (or brown bryozoans), which were natively distributed in tropical and subtropical waters and are now widespread globally through vessel hull attachment [183]. Currently, bryostatin-1 is commercially obtained by synthesis. Cancer is the main area of research of bryostatin-1 and its derivatives, with studies including applications in apoptotic restoration, multidrug-resistance circumvention, immune system stimulation, and drug synergism [184]. The low concentration of bryostatins in bryozoans (i.e., a final yield of 0.00014% after extraction) makes extraction unviable for large-scale production [143]. Moreover, the artificial breeding of bryostatin-containing organisms is of environmental concern. On the other hand, because of the complexity of its structure, total synthesis has been shown to be difficult.

### 4.9. Chitosan

Chitosan is commercially obtained mainly from chitin by the deacetylation process (enzymatic or chemical) performed by the addition of alkali solutions [112]. Chitin is very abundant in nature; this material gives strength to the exoskeletons of crustaceans, insects, and the cell walls of fungi. The main natural sources of chitin are shrimp and crab shells, which are an abundant byproduct of the food-processing industry and provide large quantities of these polysaccharides to be used in biomedical applications [112]. They can be also obtained by synthesis [145]. The extraction of chitin from the shell includes mainly demineralization and deproteinization [185,186,187]. Demineralization is carried out using concentrated hydrochloric acid (HCl) to precipitate the calcium chloride salt. The deproteinization process is often accomplished by treating the demineralized shell powder with NaOH. The demineralized and deproteinized shell powder is then subjected to decolorization by using chloroform, methanol, and distilled water and left in an oven at 60 °C for 24 h [186].

### 4.10. Meridianins

Meridianins are indole alkaloids found in marine invertebrates [147]. Several meridianins have been isolated from tunicates in recent years. For example, meridianins A–G were extracted from the species *Aplidium falklandicum* and *Aplidium meridianum* with organic solvents and further fractionated on both Sephadex LH-20 and silica gel columns. Fractions were further purified with TLC using preparative (SiO_2_) plates and HPLC using reversed-phase semipreparative C18 columns Finally, the mixture of meridianins A–G yielded 19.11 mg/g DW [148]. Similarly, meridianins F and G were isolated from the tunicate *Aplidium meridianum* [188].

**Table 2:** Main natural marine compounds with neuroprotection activity against AD, their sources, the extraction and isolation methods and yields.

Compound	Specie	Extraction/Isolation Method	Quantity	Yield (%)	Ref.
Source: Sponges					
Gracillin J	*Sponginella* sp. (Philippines) (5 g)	S-L extraction: water, MeOH/CH_2_Cl_2_ (1:1). Clean-up: Sephadex LH-20 column. Purification: reversed-phase HPLC (M.P.: MeCN/H_2_O, at 1.25 mL/min, 50 min).	9 mg	0.18%	[124]
Gracillin K			11 mg	0.22%	[124]
Gracillin L			17 mg	0.34%	[124]
Gracillin H and analog			115 mg	2.3%	[124]
Manzamine A	*Acanthostrongylophora ingens* (1.3 kg)	S-L extraction: MeOH. L-L partitioning: EtOAc and n-BuOH. Clean-up: Diaion HP-20; Sephadex LH-20. Purification: semipreparative reversed-phase HPLC. M.P.: MeOH/H_2_O/0.1% TFA.	5 mg	0.00038%	[153,154]
8-Hydroxymanzamine A			5 mg	0.00038%	[153,154]
Manzamine F			9 mg	0.00069%	[153,154]
manzamine A *N*-oxide			1.1 mg	0.000085%	[153,154]
3,4-Dihydromanzamine A *N*-oxide			1.1 mg	0.000085%	[153,154]
Source: Algae					
Fucoidan	*Sargassum siliquosum*	Pretreatment: 95% ethanol, 4 h. S-L extraction: H_2_O, 100 °C, 1 h. MAE, UAE. EtOH precipitation. Purification: anionic-exchange chromatography, dialysis,	35 mg/g	3.5%	[157]
Fucoidan	*Nizamuddinia zanardinii*	85% EtOH, RT, overnight. UAE: H2O, 55 °C, 20 kHz, 200 W, x2.CaCl_2_ precipitation, EtOH precipitation.	35.1 mg/g	3.51%	[83]
Fucoidan	*Spirarea japonica*	PLE: 0.1% NaOH, 140 °C, 50 bar. CaCl_2_ precipitation, EtOH precipitation.	11 mg/g	1.1%	[158]
Eckol, dieckol, dioxinodehydroeckol	*Sargassum fusiforme*	S-L extraction: ethanol 30% S/L ratio of 1:5) at 25 °C, 30 min. Ethyl acetate partitioning.	63.61 mg PGE/g	6.36%	[165]
Fucofuropentaphlorethol, pentafuhalol, tetrafucotetraphloretol, and PD	*Fucus vesiculosus*	S-L extraction: acetone 67% (*v*/*v*) at 25 °C. L/S ratio of 70 mL/g.	2.92 mg PGE/g DS	0.29%	[166]
Dibenzodioxine-1,3,6,8-tetrao, pentafucol, hexafucol, and PD	*Fucus vesiculosus*	MAE (ethanol 57% (*v*/*v*), of 75 °C, 5 min.	9.8 mg PGE/g DW	0.98%	[171]
Phlorotannins	*Fucus vesiculosus*	UAE (35 kHz, 30 min, 50% ethanol).	7.73 mg PGE/g	0.77%	[172]
Hydroxytetrafuhalol, triphlorethol, dihydroxypentafuhalol	*Sargassum muticuma*	PLE (60 °C and 95% ethanol).	5.018 mg PGE/g	0.5%	[167]
Eckmaxol	*Ecklonia maxima* (0.3 kg)	Sephadex LH-20 size-exclusion chromatography. HSCCC using *n*-hexane/ethyl acetate/methanol/water (2:8:3:7, *v*/*v*/*v*/*v*).	5.2 mg	0.0017%	[168]
Homotaurine	*Botryocladia leptopoda* (1 g)	S-L extraction: 10 mL ethanol 70–80% (*v*/*v*). Centrifugation (4000 rpm, 10 min). Collect the supernatant.	32.3 µg/g	0.0032%	[173,174]
Homotaurine	*Gelidium micropterum* (1 g)		32.5 µg/g	0.0032%	[173,174]
Homotaurine	*Caulerpa racemosa* (1 g)		10.56 µg/g	0.001%	[173,174]
Homotaurine	*Cystoseira indica* (1 g)		5.05 µg/g	0.0005%	[173,174]
Homotaurine	*Hypnea boergesenii* (1 g)		702.7 µg/g	0.0702%	[173,174]
Homotaurine	*Gracilaria corticata* (1 g)		474.9 µg/g	0.0474%	[173,174]
Homotaurine	*Gracilaria pygmaea* (1 g)		333.5 µg/g	0.0333%	[173,174]
Homotaurine	*Sargassum tenerrimum* (1 g)	S-L extraction: 10 mL ethanol 70% (*v*/*v*).	6.54 µg/g	0.0006%	[74]
Homotaurine	*Dyctiota dichotoma* (1 g)		14.62 µg/g	0.0014%	[74]
Homotaurine	*Gracilaria arcuata* (1 g)		6.48 µg/g	0.0006%	[74]
13-Desmethyl spirolide C and	*Alexandrium ostenfeldii* (24 cultures of 20 or 40 L)	S-L extraction: MeOH. L-L extraction: CH2Cl-H_2_O. Clean up: Sephadex LH-20. Purification: HPLC preparative: Vydac column 201TP C18, F.M. acetonitrile/water (30:70) + 0.1% TFA.	150 µg	-	[180]
13,19-Didesmethyl spirolide C			1 mg	-	[180]
Source: Bacteria					
Caniferolides A	*Streptomyces caniferus* CA-271066 (2.3 g appox)	Pretreatment: acetone. L-L extraction: ethyl acetate/water. Clean-up: Sephadex LH-20. Purification: Semipreparative reversed-phase HPLC (Agilent Zorbax RX-C8), M.P.: CH3CN/H_2_O. Semipreparative reversed-phase HPLC (XBridge C-18) (only for caniferolide D).	10.0 mg	0.43%	[25,181]
Caniferolides B			3.6 mg	0.16%	[25,181]
Caniferolides C			4 mg	0.17%	[25,181]
Caniferolides D			1 mg	0.043%	[25,181]
Caniferolide C	*Streptomyces* sp. ISID311 (1 L of culture) crude extract (2.3 g)	Pretreatment: acetone. L-L extraction: ethyl acetate/water. Clean-up: SPE-C18 (55 μm, 20 g). Purification: semipreparative HPLC.	14.2 mg	0.62%	[182]
Crustaceans					
Chitosan	Shell power (1 g)	Demineralization: 1 M HCl at 60 °C (30 min). Deproteinization: 3 M NaOH at 80 °C (120 min).	350 mg	35%	[185,186]
Meridianins A–G	*Aplidium falklandicum* and *Aplidium meridianum*	Clean-up: Sephadex LH-20 and silica gel columns. Purification: with TLC using preparative (SiO_2_) plates and HPLC (reversed-phase semipreparative C18 columns).	19.11 mg/g DW	1.91%	[148]

Abbreviatures: acetonitrile (MeCN); buthanol (BuOH); ethyl acetate (EtOAc); ethanol (EtOH); liquid–liquid (L-L); microwave-assisted extraction (MAE), methanol (MeOH); mobile phase (M.P.); phloroglucinol equivalents (PGE); phlorotannin content (PC); phlorotannin derivatives (PD); pressurized-liquid extraction (PLE); retention time (RT); solid–liquid (S-L); ultrasound-assisted extraction (UAE).

## 5. Challenges and Opportunities in the Exploitation of Marine Molecules to Manage Alzheimer’s Disease

AD constitutes one of the main social and health challenges of the XXI century with high prevalence and incidence rates, which will gradually increase due to the aging of the population [3]. Therefore, finding robust and viable lead candidates in drug discovery to manage AD is a challenging but important scientific task. At present, the benefits produced by the marine bioactive molecules to exert neuroprotection and reviewed in this paper are being considered by several research groups. Compounds such as tramiprosate or bryostatin-1 are in the clinical phase. In addition, gracilins and caniferolide A have shown relevant results [25,39]. However, testing the pharmacological effects of novel molecules is only the first step in the drug discovery process. The hardest challenge is turning these products into useful drugs due to the high costs of releasing a new medicine to market from discovery through a clinical phase to approval. Another major challenge is to reduce the time taken to approve these products for use, which is nowadays approximately 10 years [189]. In addition, the extraction, isolation, and characterization of molecules from marine sources are crucial steps of drug discovery. The industry has to invest in all these technological processes and cope with the fact that these processes do not guarantee the successful outcome of the investment. Despite growing developments in isolation methods, extraction of natural products from sponges, microalgae, and marine bacteria is still a challenging task. It has become difficult to extract and purify the scarcer novel minor secondary metabolites [151].

Comparing gracilins and manzamines, both obtained from sponges, yields from the latter are extremely low, for example, 0.00038% for manzamine A, making their purification quite unviable at a large scale. Gracilins are obtained in higher quantities ranging from 0.18 to 2.3%, and caniferolides, obtained from *Streptomyces* bacteria, are yielded in the range 0.043–0.62%. Comparing compounds from macroalgae (homotaurine, fucoidans, and phlorotannins), results revealed that it is not worth naturally extracting homotaurine. Although red algae (*Hypnea boergesenii*, *Gracilaria corticate*, and *Gracilaria pygmaea*) are the main producers of homotaurine, yields are lower than 0.0702% [173,174]. On the other hand, the extraction of fucoidans and phlorotannins from seaweeds constitutes a feasible process. It is possible to obtain fucoidans derived from Sargassum siliquosum in quantities of 35 mg/g (3.5%) [157], and phlorotannins are in levels around 63.61 mg PGE/g in *Sargassum fusiforme* (yield of 6.36%) [165]. However, to ensure progress in this field, still there is a need for determining the reproducible and well-characterized chemical composition of these molecules. For example, fucoidan fractions can vary considerably according to the natural source. These molecules can represent an enormous opportunity for the biomedical market if alternative methods to produce fucoidans with consistent properties can be developed, since these properties critically influence biological activities [190]. As said before, the low quantities of bryostatins in bryozoans (0.00014%) make extraction unviable for large-scale production [143]. On the contrary, chitosan is abundant in nature; its extraction from shells is possible, obtaining yields of 35%. Regarding the obtention of spirolides from microalgae, they can be obtained in limited amounts from contaminated shellfish, so it would be necessary to develop large-scale methods for the extraction of these toxins from phytoplankton samples [178]. In this context, an integrated approach involving high-productivity cultivation and efficient harvesting can help advance the status of microalgae technologies. Extraction of biochemical components of interest from microalgae requires cell disruption and fractionation methods. Finally, meridianins A–G are obtained in quantities of 19.1 mg/g (1.91%).

For all these molecules, the supply problem could be solved by processes of marine biotechnology (aquaculture/mariculture/fermenter cultivation, genetic engineering, enzymatic synthesis or modification) or by chemical synthesis/semisynthesis/modification [191]. In addition, the development of new extraction technologies has revolutionized the screening of natural marine products (specially macroalgae) in discovering new drugs. Applying these technologies offers a unique opportunity to re-establish natural products as a major source for drug discovery. Alboofetileh et al. employed conventional and different innovative extractions, using water as the solvent, to obtain fucoidans from *Nizamuddinia zanardinii*. Conventional extraction showed an extraction yield of 5.2% (DW). MAE and PLE achieved higher rates, 6.17% and 13.15%, respectively [83]. The emerging extraction techniques display some advantages when extracting secondary metabolites from cells. The growth and collapse of the bubbles created in UAE break the cell walls and particles, improving the extraction by increasing the contact between the sample and the solvent. It works at low temperatures, which allows the preservation of thermolabile compounds [85,157,162]. MAE, based on the use of microwaves to heat the samples, causes the evaporation of the intracellular fluids, providing an increase in pressure that breaks the cell and consequently releases intracellular compounds into the solvent. Compared to S-L extraction, MAE reduces the amount of solvent necessary and improves efficiency [84,85]. PLE uses less solvent, is performed in a shorter period, is automated, and involves retaining the sample in an oxygen and light-free environment in contrast to traditional organic solvent extraction [192]. Finally, SCFE brings some advantages. When a fluid is taken above its critical temperature and pressure, it exists in a condition referred to as a supercritical state. For example, carbon dioxide, once pressurized, can act as an effective solvent of nonpolar compounds. SCFE is finding increased and widespread use in the extraction from solids and liquids of such compounds with novel use as nutraceuticals and pharmaceuticals.

## 6. Remarks and Future Perspectives

This review explores the use of sponges, algae, and other marine species to obtain molecules of high potential value with evidence-based health benefits against AD and their isolation by chromatographic and emerging extraction techniques. A critical point in the process of drug development from marine organisms and often a bottleneck is the need to obtain permanent availability of sufficient amounts of organisms and compounds without harming the marine environment. Only if supply can be addressed in an economically and ecologically feasible fashion will marine drugs reach the market [191]. The reviewed literature shows that macroalgae constitute a cost-effective and useful source for recovering phlorotannins and fucoidans with high potential for innovative biomedical applications. Chitosan has also attracted a great deal of attention in health applications due to the distinctive pharmacological and physicochemical characteristics of this molecule, which can be extracted from crustacean waste and contribute to a sustainable recovery process. In this context, environmentally friendly processes that combine various microbial, chemical, enzymatic, and membrane strategies and technologies to extract and purify chitin and chitosan from marine byproducts were also investigated [193].

One of the most promising molecules to manage AD is bryostatin-1, which is in the clinical phase. However, synthetic molecules are used as the supply for clinical trials. In preclinical studies, bryostatin-1 was found to improve memory and learning for prolonged periods, produce neuroprotective effects in transgenic mice with AD, inhibit phosphorylation of the tau protein by inhibiting the enzyme GSK3beta, prevent neuronal apoptosis, decrease amyloid-beta levels in the Tg 2576 mouse with AD, recover neurotrophic activity and loss of synapses, and improve synaptogenesis, recovering cognitive deficits [119,120,183,194,195].

Besides these compounds, gracilins and caniferolide A, obtained from natural sources (*Sponginella* sp. sponges and *Streptomyces caniferus*, respectively), have shown satisfactory results. After chronic intraperitoneal treatment of gracilins in 3xTg-EA mice, it was observed that gracilins H and L can produce an improvement in cognition [39]. Gracilin L is the only one capable of inhibiting the BACE-1 enzyme in vitro and *in vivo*, and gracilins H and L decrease the levels of Ab42 and phosphorylated tau protein *in vivo*. In general, gracilins reduce phosphorylation of tau protein by inhibiting ERK in vitro [27,39,125,127]. Parallelly, caniferolide A modulates the state of the microglial cell towards the phenotype of neuroprotection by causing the blockade of the kinases NFKR, p38, and JNK MAPK. This macrolide was also able to decrease the phosphorylation of the tau protein by inhibiting the p38 and JNK kinases, and through the translocation of Nrf2 to the nucleus, it acts as an indirect antioxidant [25,182]. This compound is also capable of inhibiting the BACE-1 enzyme, which participates in the processing of amyloid-beta.

The oceans can therefore provide us with many invaluable benefits and services, including some of the medicines we use to manage AD. Even so, research on compounds of marine origin for the treatment of AD should continue in the search for new drugs with significant neuroprotective activity. A better understanding of the potential health benefits from marine organisms, the compounds they produce, and the environmental conditions affecting their production will allow for the better management and sustainable development of these valuable marine resources in the future [189].

Although great advances have been achieved in a short time in discovering new molecules, the riskiest development stages of a potential drug (production optimization, safety studies, and clinical trials) are assumed by the academic world or small biotechnology laboratories. To continue to give answers to human needs, more investment in biotechnology should be encouraged and stimulated.

## Figures and Tables

**Table 1:** Main natural marine compounds with pharmacological activity to treat AD.

Compound	Origen	Family	Mechanism of Action	Ref.
Sponges				
Gracilins	Marine sponges (*Spongionella gracilis*)	Diterpenoid derivatives	Inhibition of the enzyme b-secretase or BACE-1. Anti-inflammatory and antioxidant properties. Reduction in hyperphosphorylation of tau protein.	[39]
Manzamines	Marine sponges (*Haliclona* sp.	Alkaloids with beta-carboline structure	Inhibition of GSK3beta and CDK5.	[114]
Macroalgae and microalgae				
Fucoidans	Brown seaweeds	Sulfated polysaccharides	Block caspase-9 and caspase-3 enzymes.	[115]
Phlorotannins	Brown seaweeds (*Ecklonia cava*, *Ecklonia stolonifera*)	Polyphenols	Inhibition of the enzymes acetylcholinesterase and butyrylcholinesterase.	[116]
Homotaurine	Red seaweeds	Aminosulfonate	Prevention of the formation of a toxic soluble amyloid oligomer.	[117]
Spirolides	*Alexandrium ostenfeldii/peruvianum* dinoflagellates	Cyclic imines	Decrease GSK-3β and ERK in 3xTg mice cortical neurons. Glutamate-induced neurotoxicity inhibition both in control and 3xTg neurons.	[118]
Bacteria				
Caniferoles	Phylum Actinobacteria	Polyol macrolides	Anti-inflammatory and antioxidant action. Blockade of the BACE-1 enzyme.	[25]
Marine invertebrates, crustaceans, tunicates				
Bryostatins	Brown bryozoa (*Bugula neritina*)	Macrolide lactones	Modulates neuronal synapses under synaptic dysfunctions; improvement of memory, cognition, and spatial learning; decreases amyloid-beta peptide; reappearance of neurotrophic activity.	[119,120]
Chitosan	Crustaceans	Polysaccharides	Inhibition of the enzyme acetylcholinesterase.	[121]
Meridianins	Tunicates (*Aplidium meridianum*)	Alkaloid indols	Inhibition of GSK3beta, CK1sigma, DYRK1A, and CLK1.	[122,123]

Abbreviations: beta-secretase enzyme (BACE); cyclin-dependent kinase 5 (CDK5); glycogen synthase kinase 3 beta (GSK-3β); extracellular signal-regulated kinase (ERK); dual-specificity tyrosine phosphorylation-regulated kinase 1A (DYRK1A); cell division cycle 2-like kinase 1 (CLK1).

## Data Availability

Not applicable.
